# Porous Layer-by-Layer Films Assembled Using Polyelectrolyte Blend to Control Wetting Properties

**DOI:** 10.3390/polym13132116

**Published:** 2021-06-28

**Authors:** Choonghyun Sung, Yejin Heo

**Affiliations:** Division of Advanced Materials Engineering, Dong-Eui University, Busan 47340, Korea; jim58061@naver.com

**Keywords:** layer-by-layer, porous, wetting, roughness, polyelectrolyte multilayer, slippery

## Abstract

Porous layer-by-layer (LbL) films have been employed for the implementation of superwetting surfaces, but they are limited to the LbL films consisting of only two oppositely charged polyelectrolytes. In this study, LbL films were assembled using a cationic polymer blend of branched poly(ethylene imine) (BPEI) and poly(allylamine hydrochloride) (PAH), and anionic poly(acrylic acid); they were then acid-treated at pH 1.8–2.0 to create a porous structure. The films of 100% BPEI exhibited a relatively smooth surface, whereas those of the 100% PAH exhibited porous surfaces. However, various surface morphologies were obtained when BPEI and PAH were blended. When coated with fluorinated silane, films with 50% and 100% PAH exhibited relatively higher water contact angles (WCAs). In particular, films with 50% PAH exhibited the highest WCA of 140–150° when treated at pH 1.8. These fluorinated films were further infused with lubricant oil to determine their feasibility as slippery surfaces. The water and oil sliding angles were in the range of 10–20° and 5–10°, respectively. Films prepared with the BPEI/PAH blend showed lower water slide angles than those prepared with 100% BPEI or PAH. Acid treatment of LbL films assembled using a polyelectrolyte blend can effectively control surface morphologies and can potentially be applied in superwetting.

## 1. Introduction

Extensive research has been conducted on superwetting surfaces as they play a crucial role in solving numerous engineering problems [1,2,3,4]. Superwetting surfaces are surfaces with extreme wettability, such as superhydrophobic, superhydrophilic, and omniphobic surfaces. They have been utilized in electronic, biomedical, energy, and environmental applications owing to their unique properties such as self-cleaning [5], anti-icing [6], oil-repellency [7], and anti-fouling [8]. The analysis of plant or animal surfaces with special wettability serves as a catalyst for the artificial synthesis of superwetting surfaces. Extensive research has been conducted on superhydrophobic surfaces owing to the discovery that the self-cleaning capability of lotus leaves originates from micro/nano hierarchical structures with low surface energy [9]. There have been many reviews concerning superhydrophobic surfaces [2,10], one of which includes many patents [10]. Researchers have synthesized an omniphobic slippery liquid-infused porous surface (SLIPS) based on the characteristics of the *Nepenthes* pitcher plant, which captures insects by using the slippery surface where nectar is locked in the microscale texture [11,12]. The Aizenberg group has developed many SLIPS and published many patents concerning their practical applications [13,14,15].

The micro/nano hierarchical structure or proper surface texture is crucial in the implementation of superwetting surfaces. Consequently, research has been focused on the fabrication of proper surface textures using various techniques such as sol-gel [16], lithography [17], electrodeposition [18], electrospinning [19], and hydrothermal methods [20]. However, these techniques often use toxic chemicals, are complicated and difficult to scale up, and cannot be easily applied to curved surfaces. Recently, the layer-by-layer (LbL) assembly technique has been actively employed in the fabrication of superwetting surfaces [21]. This technique is used to fabricate thin films on substrates of any shape in a simplified manner by alternately immersing a substrate in aqueous solutions of oppositely charged materials, such as polymers, surfactants, and nanoparticles [22,23]. The film thickness, structure, and other properties of the LbL films are controlled by the material chemistry and the assembly conditions.

The incorporation of nanoparticles is one of the most frequently used methods to build superhydrophobic surfaces, which are commonly fabricated by using the LbL assembly of polyelectrolytes and nanoparticles, followed by coating with a low surface energy material [24,25]. In another study, a polyelectrolyte/nanoparticle LbL film was thermally decomposed to yield a nanoparticle-only porous film [26]. The other methods used to build superhydrophobic surfaces involves employing polyelectrolyte complexes such as polyelectrolyte-polyelectrolytes [27,28] or polyelectrolyte-surfactant complexes [29]. These complexes enable the rapid growth of micrometer-thick LbL films with rough surfaces.

Alternatively, porous LbL films have been employed in the fabrication of superhydrophobic surfaces. For the preparation of porous LbL films, the post-assembly treatment of LbL films built from weak polyelectrolytes has been extensively analyzed. Although the basic treatment [30] and electrochemical methods [31] have been reported, most studies have been focused on the acidic treatment of the LbL films of poly(allylamine hydrochloride) (PAH)/poly(acrylic acid) (PAA) [32,33] or poly(ethyleneimine) (PEI)/PAA [34]. The first superhydrophobic surfaces prepared using the LbL assembly technique were fabricated by a two-stage acid treatment of the PAH/PAA LbL film followed by nanoparticle deposition and silanization [35]. Microscale roughness was obtained from the porous structure, and nanoscale roughness was obtained from the nanoparticles. In another study, a superhydrophobic surface was prepared by the synchronous generation of nano/microscale hierarchical porous structures in the LbL films without nanoparticle deposition using a combination of high-molecular weight (MW) PAH and low-MW PAA with a short deposition time of 1 min [36]. Recently, porous PAH/PAA LbL films were fabricated using the PAA with a bimodal MW distribution [37,38] based on the fact that the porous structure is influenced by the PAA MW in the PAH/PAA LbL film [39,40,41]. It was demonstrated that the surface morphologies and the wetting properties of the porous LbL films could be controlled by adjusting the blend ratio of low- and high-MW PAAs. 

Porous LbL films have also been used in the fabrication of SLIPS. Huang et al. utilized the porous BPEI/PAA LbL films fabricated by two-stage acid treatment, along with the deposition of silica nanoparticles for SLIPS [42]. The Aizenberg group prepared SLIPS by using a porous film prepared by the calcination of a polyelectrolyte/silica LbL film [43]. Shiratori et al. utilized the porous LbL films of chitin nanofiber/silica nanoparticles in the implementation of SLIPS [44]. Additionally, the porous LbL films prepared by using azlactone polymers [45] and hierarchically rough PEI/Nafion LbL films assembled in organic solvents [46] have been used as porous matrices for SLIPS. Furthermore, the microstructure with a worm-like surface morphology obtained from the pH-amplified exponential growth of the PEI/PAA film, was used for the patterned SLIPS, which can guide the sliding movement of liquid droplets [47].

Previous studies have focused on acid treatment conditions to generate porous structures in LbL films. In these studies, either a time-consuming two stage acid treatment was applied to fabricate nano/micro hierarchical structure or additional nanoparticles were deposited on the porous LbL films. This implies that generating the hierarchical or complex structure is difficult because a pore formation mechanism is determined by one pair of cationic/anionic polyelectrolytes. In this study, porous LbL films were prepared by using a cationic polyelectrolyte blend. When the two cationic polyelectrolytes are used in the LbL assembly, two pore formation mechanisms from two pairs of cationic/anionic polyelectrolytes operate during post-assembly acid treatment. Therefore, it provides another method of fabricating various complex pore structures in LbL films. 

Here, we present porous LbL films consisting of the branched PEI (BPEI), PAH, and anionic PAA. Although the PEI/PAA or PAH/PAA LbL films have been used separately in the preparation of porous structures, the BPEI/PAH blend has not been utilized thus far. PAH is a linear polymer with a pK_a_ value of 8–9 in solution [48], while BPEI is a branched polymer that contains primary, secondary, and tertiary amine groups with pK_a_ values ranging from 6–9 [49]. Therefore, even though the assembly pH and the post-assembly acid treatment conditions are identical, the porous structures of the BPEI/PAA and PAH/PAA LbL films vary. Consequently, the LbL films were prepared in this study by using BPEI/PAH solutions with various BPEI/PAA blend ratios, and the films were acid-treated at pH 1.8–2.4. Various surface morphologies and porous structures were obtained based on the BPEI/PAH blend ratio and the pH of the acid treatment. These films were further coated with fluorinated silane, and the water and oil contact angles were measured. Moreover, the fluorinated samples were infused with a fluorinated lubricant to obtain a slippery surface, and the wetting properties and sliding performance were analyzed.

## 2. Materials and Methods

### 2.1. Materials

The cationic polymer, branched polyethyleneimine (BPEI, MW = 760,000 g/mol), and poly(allylamine hydrochloride) (PAH, MW = 160,000 g/mol) were purchased from Aldrich (St. Louis, MO, USA) and Alfa Aesar (Haverhill, MA, USA), respectively. The anionic polymer poly(acrylic acid) (PAA, MW = 100,000 g/mol) was purchased from Aldrich. Trichloro (1H, 1H, 2H, 2H-perfluorooctyl silane) (TCPFS, 97% purity) was obtained from Aldrich (St. Louis, MO, USA). The fluorinated lubricant, Krytox GPL101 (95% purity), was obtained from Chemours (Wilmington, DE, USA). Acetone and methanol of 99.5% purity were purchased from Samchun Chemicals (Seoul, Korea).

### 2.2. Fabrication of LbL Film

The LbL films were assembled using a programmable multiple dip-coater (HT-17, Hantech, Daejeon, Korea). The glass slides were cleaned by sequential sonication in acetone, methanol, and deionized water for 10 min each. These slides were oxygen plasma-cleaned for 2 min immediately before the LbL assembly. The slides were then dipped into the cationic solution for 15 min and rinsed three times (for 2 min, 1 min, and 1 min) in deionized water. A blend of BPEI and PAH was used as the cationic solution. The cationic solution was prepared by blending 1 g/L BPEI and 1 g/L PAH solution at five volume ratios of BPEI:PAH = 100:0, 75:25, 50:50, 25:75, and 0:100. The PAH ratio was used for determining the ratio of BPEI and PAH in the mixture ratio; for example, 25% PAH refers to BPEI:PAH = 75:25. The pH of the solution was adjusted to 7.5. The BPEI/PAH solution is denoted as the BPAH solution. These glass slides were then dipped into 1 g/L PAA solution (pH 3.5) for 15 min and rinsed three times in water, as described previously. This process was repeated to yield 20.5 bilayers, that is, (BPAH/PAA)_20_BPAH. The samples were dried in an oven at 80 °C for 30 min.

### 2.3. Post-Assembly Acid Treatment and Surface Coating

The LbL films assembled on glass slides were immersed in acidic solutions (pH 1.8, 2.0, 2.2, and 2.4) for 1 h. The samples were rinsed with deionized water for 2 min. They were then dried in an oven at 80 °C for 30 min, followed by heating at 180 °C for 2 h to crosslink the LbL film [32,36]. To prepare the hydrophobic LbL film, the acid-treated samples were coated with fluorinated silane via chemical vapor deposition under atmospheric conditions. The samples were placed in a 150 mL sealed bottle containing 90 μL of TCPFS, and left at room temperature for 6 h. The slippery surfaces were prepared by infusing a fluorinated lubricant, GPL101, on the TCPFS-functionalized LbL film. 100 μL of GPL101 was spread over the film surface. After waiting for 1 min, the film was spin-coated at 2000 rpm for 1 min. 

### 2.4. Characterization

The thickness and the root mean square (RMS) roughness of the LbL films were measured using a profilometer (Alphastep D-100, KLA-Tencor, Milpitas, CA, USA). The surface morphologies were analyzed by using a scanning electron microscope (SEM; Quanta 200 FEG, Thermo Fisher Scientific, Waltham, MA, USA) at Core-facility for Converging Materials of Dong-eui University. An 8 nm thick platinum layer was sputtered onto the film before measurement. The contact angle (CA) and sliding angle (SA) of water and hexadecane oil were measured using a CA meter (Phoenix-150, SEO, company, Suwon, Korea) equipped with an automatic tilt stage. Droplets of 3 µL and 15 µL were used for the CA and SA measurements, respectively. The tilt angle was increased from 0° to 50°, and the tilt angle at which the droplet began to slide was recorded as the slide angle.

## 3. Results and Discussion

**Figure 1:**
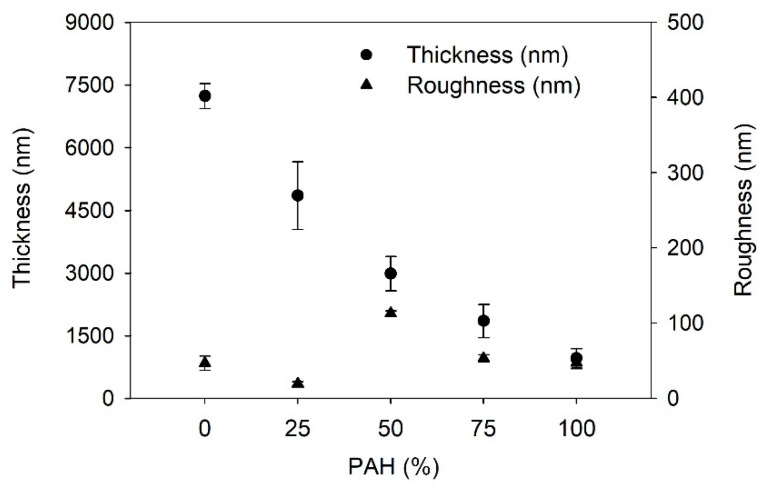
Thickness and roughness of as-prepared LbL film as a function of PAH ratio.

Firstly, the growth of the LbL films is determined; Figure 1 shows the film thickness and the roughness of the LbL films as a function of the PAH ratio prior to the acid treatment. With the increase in the PAH ratio, the thickness of the LbL film decreased from 7200 to 960 nm. BPEI is a branched polymer and PAH is a linear polymer. Furthermore, the average pK_a_ of BPEI is lower than that of PAH. Thus, the degree of ionization of BPEI is lower than that of PAH. It has been reported that the degree of ionization of BPEI and PAH is 20% and 80%, respectively at the assembly pH of 7.5 [42,48]. Thus, the conformation of BPEI is more coiled than that of PAH during the adsorption on the film surface. Therefore, the films with higher amounts of BPEI will be thicker. These results confirm that the composition of the LbL film varies based on the PAH ratio of the polymer solution used in the LbL assembly. The surface roughness was approximately below 50 nm, although it showed the highest value of 113 nm when the PAH ratio was 50%. It is unclear why the roughness is highest at 50% PAH. One possible explanation is that an even mixture of two polymers caused a structural difference in the film compared to the other films with different PAH ratios.

**Figure 2:**
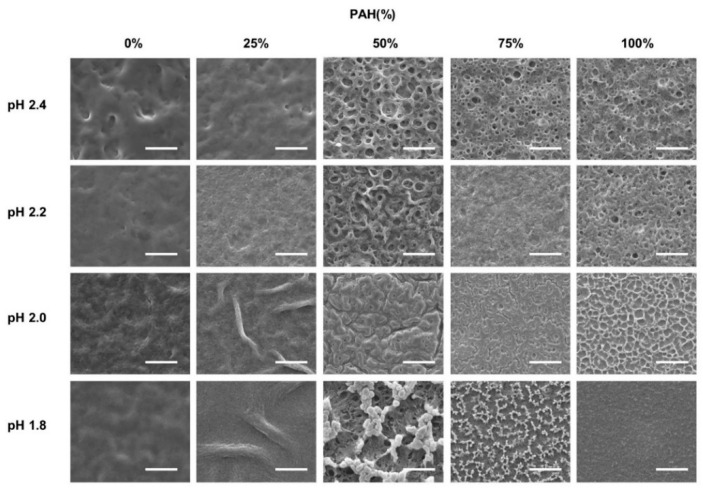
SEM images of LbL films of varying BPEI/PAH ratios acid-treated at varying pHs. Acid-treatment time was fixed at 1 h. The scale bars are 20 μm.

**Figure 3:**
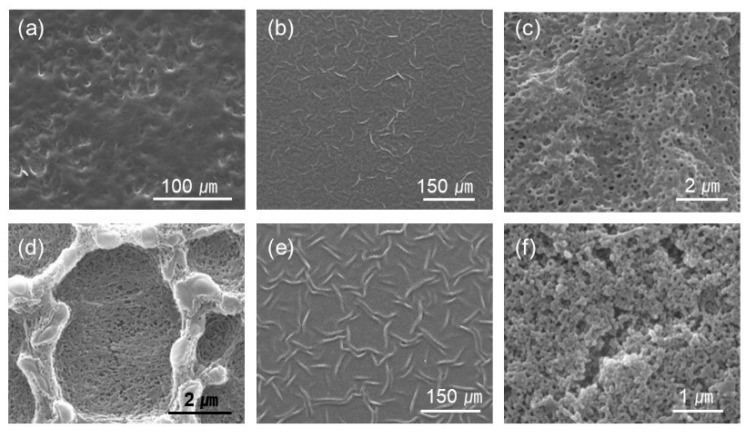
Selected images of different magnification from Figure 2. The PAH ratio and acid treatment pH are (**a**) PAH 0%, pH 2.4 (**b**,**c**) PAH 25%, pH 2.0, (**d**) PAH 100%, pH 2.0, (**e**) PAH 25%, pH 1.8, and (**f**) PAH 100%, pH 1.8, respectively.

The as-prepared LbL films with five BPEI/PAH ratios were further immersed in acidic solutions (pH 1.8–2.4) for 1 h to generate a porous structure. Figure 2 shows the surface morphologies of the acid-treated LbL films analyzed with SEM at a magnification of 3000×. Some of the samples were examined at different magnifications, as shown in Figure 3. The effect of the PAH ratio on the surface morphology at a given acid treatment pH is discussed, referring to Figure 2, and Figure 3 is used as supplementary materials to Figure 2.

The surface was fairly smooth for the films with 0% PAH ratio (100% BPEI) at pH 2.4, but rough surfaces were observed at a lower magnification (Figure 3a)**.** As the PAH ratio increased over 50%, a clear microporous structure was observed on the film surface, and the pore size was larger for the films with a 50% PAH ratio. At pH 2.2, the largest and the most distinct pore structures were also observed at a PAH ratio of 50%. However, the film surface was relatively smooth at a 75% PAH ratio, but a distinct porous structure was observed again at 100% PAH ratio. 

At pH 2.0, the LbL films exhibited slightly different morphologies. When the PAH ratio was 0%, the film exhibited a relatively smooth surface. However, as the PAH ratio increased to 25%, the LbL films exhibited wrinkles with nanopores, as shown in Figure 3b,c. Conversely, for the films with 100% PAH ratio, the well-known microscale crater-like pore structure was observed; nanopores were also observed inside the crater at higher magnifications (Figure 3d). However, as the PAH ratio decreased from 100% to 50%, the thickness of the wall increased and the size of the crater decreased, and nanopores were detected in the valley.

At pH 1.8, when the PAH ratio was 0%, the surface was relatively smooth. As the PAH ratio was increased to 25%, wrinkles with nanopores were detected, which were observed for the samples treated at pH 2.0. However, the wrinkles became larger and thicker (Figure 3e). Conversely, when the PAH ratio was 100%, the film surface was relatively smooth. The crater-like pores or micropores were no longer detected, but small aggregates were observed at higher magnifications (Figure 3f). This is due to the partial dissolution of the film and will be explained in the later sections of the paper. However, as the PAH ratio decreased from 100% to 75%, small crater-like pores were observed, and these crater-like pores were largest at a PAH ratio of 50%. Additionally, smaller pores or crevices were also detected inside the craters.

Overall, when the BPEI ratio was 100%, there were no distinct features, and when the PAH ratio was 100%, a porous structure was developed. However, various surface morphologies and pore structures appeared as BPEI and PAH were blended.

**Figure 4:**
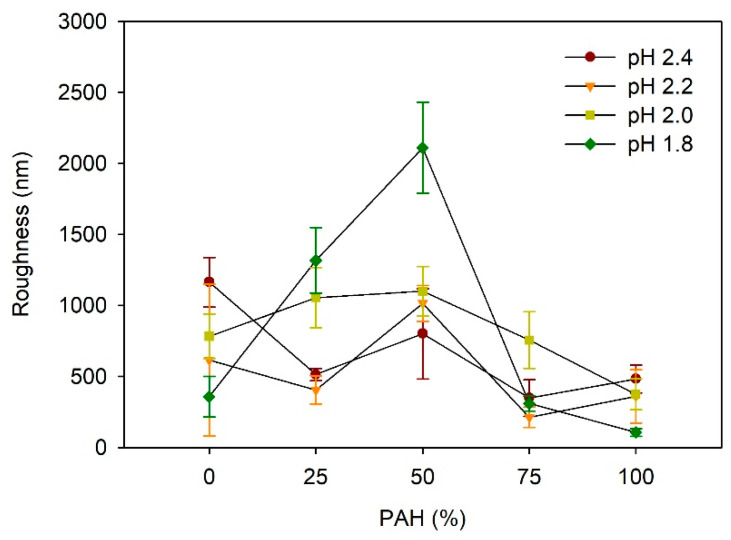
Influence of PAH ratio on the roughness of LbL films treated at different pH values.

Figure 4 depicts the effect of the PAH ratio on the LbL film roughness after acid treatment at various pH values. At pH 2.4, the roughness was highest for the films with a PAH ratio of 0%, which is approximately 1164 nm. The higher roughness of the samples with 0% PAH despite the lack of a porous structure is due to the larger scale of the roughness, as shown in Figure 3a. At a PAH ratio of 25%, the roughness decreased to 514 nm, but it increased to 800 nm at a PAH ratio of 50%, owing to the porous structure. Although a porous structure was observed in the films with 75% and 100% PAH, the roughness decreased slightly. At pH 2.2, the maximum roughness value of 1014 nm was observed at a 50% PAH ratio. This result is consistent with the surface morphology analyzed with the SEM, where the pores were most distinct and largest at a PAH ratio of 50%. At a PAH ratio of 75%, the roughness decreased to 213 nm, which is consistent with the smooth surface shown in the SEM images. At a PAH ratio of 100%, the roughness increased slightly to 361 nm owing to the porous structure, as shown in Figure 2. 

At pH 2.0, the roughness increased slightly from 783 nm to 1100 nm as the PAH ratio increased from 0% to 50%, and it decreased to 375 nm as the PAH ratio increased further to 100%. The samples with 0% and 25% PAH did not exhibit a porous structure, and the higher roughness was attributed to the large-scale irregular surface texture, similar to the images shown in Figure 3a. Contrarily, the samples of 50–100% PAH exhibited relatively low roughness, even though a porous structure or surface texture was observed in the SEM images. As the PAH ratio increased from 50% to 100%, the roughness decreased due to the decrease in the pore size. 

At pH 1.8, the influence of the PAH ratio on the roughness was the greatest. The roughness showed the highest value of 2109 nm at a PAH ratio of 50%, where a large crater-like pore structure was observed. Similarly, the roughness decreased in tandem with the further variation of the PAH, owing to the weakening or the disappearance of the microporous structure. Overall, the dependence of the roughness on the PAH ratio and acid treatment pH is consistent with the SEM images of the surface morphologies.

**Figure 5:**
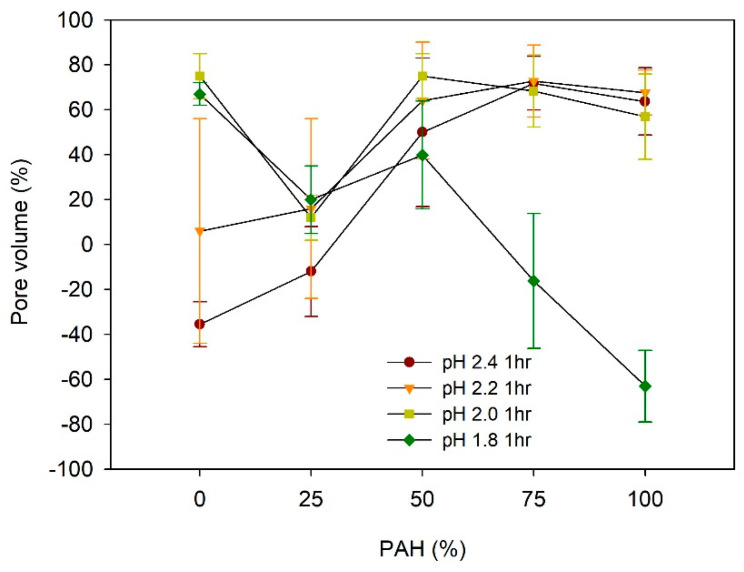
Pore volume of LbL films as a function of PAH ratio and acid treatment.

The pore volume (%) of the acid-treated LbL films is estimated from (H − H_0_)/H × 100, where H is the film thickness after acid treatment and H_0_ is the film thickness before treatment, assuming no change in the mass [27,29]. Figure 5 describes the dependence of the pore volume on the PAH ratio and pH of the acid treatment. 

For the films assembled with 0% PAH ratio, the pore volume increased from −35% to 75% with the decrease in the pH, from pH 2.4 to pH 1.8. The negative value of the pore volumes at pH 2.4 is attributed to the loss of mass from the film [34,42]. The pore volume at pH 2.2 was 6%, but it showed a large deviation. This is because pH 2.2 is the transition pH above which a large pore volume is observed. At pH 2.2, one region showed pores and the other region showed film dissolution in the same sample, as explained later in the paper. The pore volume changes to a large positive value at pH 2.0 and 1.8, which indicates a clear porous structure. This indicates that there are pores beneath the surface although the film surface is fairly smooth, as shown in Figure 2. This “skin layer” above the pore structure has been observed for linear PEI (LPEI) /PAA LbL films assembled at pH 5 [34] or the PAH/PAA LbL films assembled with low MW PAA [39,40,41]. Its origin remains unclear, but it has been attributed to the higher mobility of the polyelectrolyte in the exponentially grown LbL films. It has been demonstrated that the PEI has higher mobility than the PAH [50,51], and the low-MW PAA is estimated to have better mobility than the high-MW PAA [40]. 

In contrast, when the film was assembled with the 100% PAH, the pore volumes were 55–70% when treated at pH 2.0–2.4. However, the pore volume became −63% when the pH was 1.8, indicating the loss of mass. This pH dependence of the pore volume for 100% PAH films is consistent with previous studies [32,33,36]. 

Considering the dependence of the pore volume on the PAH ratio, at pH 2.2–2.4, the pore volume increased with the increase in the PAH ratio from 0% to 50%. However, the pore volume did not vary significantly above a PAH ratio of 50%. Conversely, at pH 1.8, the pore volume decreased with the increase in the PAH ratio. 

**Figure 6:**
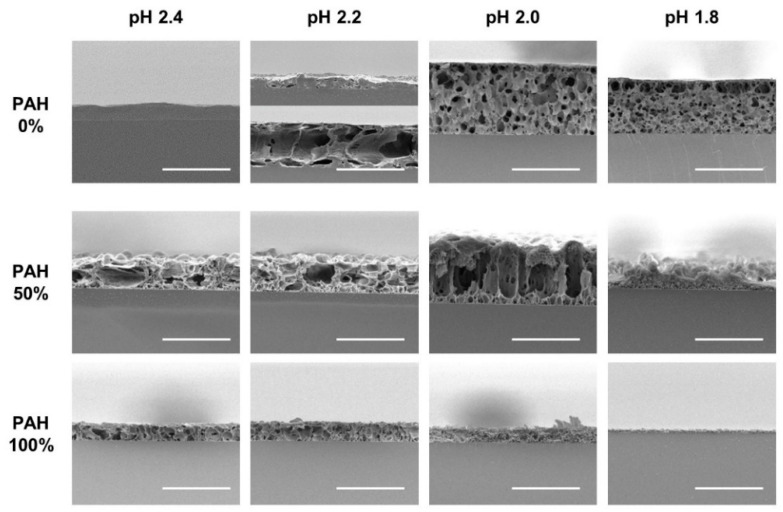
Cross-sectional SEM images of LbL films assembled with the 0%, 50%, and 100% PAH ratio treated at different pH values. Two regions with different structures were observed for the film assembled with 0% PAH ratio treated at pH 2.2 (The 2nd image in the first row). The scale bars are 20 μm.

To verify the pore structure of the acid-treated films, cross-sections of the selected films were analyzed with SEM (Figure 6). The first row displays the acid-treated films with a 0% PAH ratio. At pH 2.4, the films were partially dissolved without pores in most regions. At pH 2.2, the samples began to show a pore structure; however, it was inhomogeneous. In one region, the film was thin, with a few pores owing to the film dissolution, while in the other region, the film was thick, with large pores. This is because pH 2.2 is the transition pH for the pore formation. At pH 2.0 and 1.8, microporous structure is observed homogeneously beneath the “skin layer”. Contrarily, the 100% PAH films showed a clear pore structure at pH 2.0–2.4, but the pore sizes are smaller than those of the films with 0% PAH. At pH 1.8, the dissolution of the film was observed, as shown in the third row of the figure. Furthermore, the LbL films assembled with a 50% PAH ratio showed larger pore sizes when compared to the films assembled with 0% PAH due to the BPEI blending. 

At an acid treatment pH of 2.4, the films of the 0% PAH exhibited film dissolution (−40% pore volume), in contrast to the PAH/PAA films where a porous structure with increased thickness was detected. Similar results were observed for the other PEI/PAA films. A decrease in the film thickness has been observed for the PEI/PAA films assembled with partially ionized PEI and PAA. The LPEI/PAA films assembled at pH 3 and treated at pH 2.5, showed −20% pore volume, while those assembled at pH 4–5 and treated at pH 2.5, showed 40–60% pore volume [34]. The BPEI/PAA films assembled with BPEI at pH 6.5–9.5, and PAA at pH 4.5, showed large distinct pores but with a 20% decrease in the film thickness when treated at pH 3.0 [42]. As the acid treatment pH decreased further to pH 2.0, the films showed an 80% decrease in the thickness with fine pores. 

However, for the films with 0% PAH used in this study, which were assembled with BPEI at pH 7.5 and PAA at pH 3.5, the pore volume increased from −40% to 80% with a microporous structure as the acid treatment pH increased above 2.0. This is assumed to be because of the film structure with higher interdiffusion and chain entanglement. The degree of ionization in solution is below 10% and 20% for PAA and BPEI at the assembly, pH 3.5 for the PAA and pH 7.5 for the BPEI [42,46]. At this low degree of ionization, the LbL films typically consist of loop-rich intermingled structures because of the more coiled polymer chain conformation [32,52]. At an acid treatment pH of 2.4, the BPEI ionization and PAA protonation might be insufficient for film swelling, but sufficient for slow film dissolution, which can be attributed to the higher mobility of the BPEI chains, as reported in the previous studies conducted on interdiffusion [50,51]. However, at an acid treatment pH of 1.8–2.0, the BPEI ionization and PAA might be sufficient for the significant film swelling overcoming the intermingled structure. 

Considering the above results, the effects of PAH ratio and acid treatment pH on film morphologies and roughness are explained as follows. At pH 2.4 and 2.2, the 0% PAH film partially dissolved, which caused the film to have a rough surface. In contrast, the 100% BPEI film showed a microporous structure with reduced roughness. When BPEI and PAH were evenly mixed, the films formed a porous structure with partial film dissolution. In this process, larger pores were formed on the film surfaces. At pH 2.4, the roughness showed values between those of the 100% BPEI and 100% PAH film, where an increase in pore size is not very high. At pH 2.2, the highest roughness value was obtained as larger pores were formed.

At pH 2.0, the 100% BPEI films exhibited large pores in their structures beneath the skin layer, while the 100% PAH films showed a crater-like porous structure. At 50% PAH ratio, PAH contributed to the removal of the skin layer and different pore structures appeared owing to the simultaneous operation of the two pore generation mechanisms. Accordingly, maximum roughness was observed at 50% PAH ratio. 

At pH 1.8, the 100% BPEI films also exhibited large pores in their structure beneath the skin layer, but the 100% PAH films showed partial film dissolution. As BPEI and PAH were mixed, the skin layer was removed due to the PAH contribution, and larger crater-like pores were developed owing to the synergistic effect of film dissolution and pore formation. Moreover, the highest roughness value was obtained at the 50% PAH ratio.

**Figure 7:**
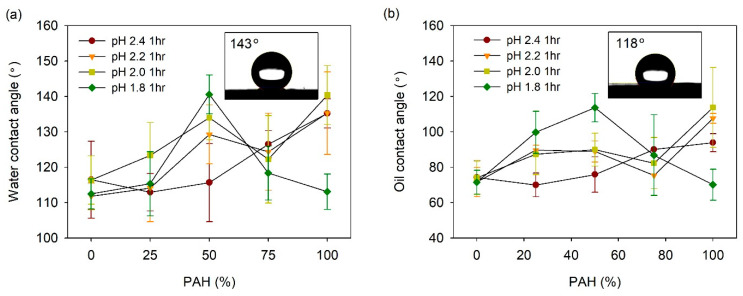
(**a**) WCA and (**b**) OCA of acid-treated LbL films coated with TCPFS. Inset figures in Figure 7a,b show water and oil droplet on 50% PAH film treated at pH 1.8, respectively.

The acid-treated LbL films prepared with the BPEI/PAH blends were coated with fluorinated silane, 1H, 1H, 2H, 2H-perfluorooctyl silane (TCPFS), and the water and oil CAs were measured. Figure 7a shows the water contact angle (WCA) vs. the PAH ratio plot at a given acid treatment pH. At pH 2.4, the WCA was 110–117° for the PAH ratio of 0–50%. However, as the PAH ratio increased from 50% to 100%, the WCA increased from 116° to 140°, although the roughness decreased slightly with the PAH ratio in this range. This indicates that an appropriate porous structure is an important factor for high WCA when compared to the roughness alone. 

At pH 2.2, the WCA showed higher values at the PAH ratios of 50% and 100% when compared to the other PAH ratios. These are the ratios at which a clear pore structure was observed on the surface, as shown in Figure 2. The dependence of the WCA on the PAH ratio at pH 2.0, was similar to the result observed at pH 2.2. Overall, the WCA was relatively high at the PAH ratios of 50% and 100%, where the surface porous structure was distinct. At pH 2.0, the film roughness was highest at the 50% PAH ratio, whereas it was lowest at the 100% PAH ratio. However, the WCA was the highest at the 100% PAH ratio. Although the roughness was lowest at this PAH ratio, the microscale crater-like structure with nanopores is assumed to have caused the high WCA. 

The effect of the BPEI/PAH blending was the greatest at pH 1.8. The highest WCA value of 141° was observed for the 50% PAH film treated at pH 1.8, which exhibited the highest roughness and largest crater-like pore structure with inner nanopores. The WCA decreased to 113° with the further variation of the PAH ratio from 50%. This is due to the disappearance of the porous structure on the film surface. 

The oil contact angle (OCA) of the LbL films was also analyzed (Figure 7b). In general, the influence of the PAH ratio on the OCA was similar to the result obtained for the WCA. At pH 2.4, the OCA increased from 74° to 94° with an increase in the PAH ratio. At pH 2.2 and 2.0, the OCA was relatively high at the PAH ratios of 50% and 100%. The highest value of the OCA was 108–114° at the PAH ratio of 100%. At pH 1.8, the OCA exhibited the highest value of 114° at a PAH ratio of 50%. Therefore, it was possible to control the WCA and OCA of the film by varying the PAH ratio and the acid treatment pH.

The highest values of WCA and OCA obtained for PFTCS-coated LbL film were 141° and 115°, respectively. These values were obtained for films with 50% PAH ratio treated at pH 1.8 and those with 100% PAH ratio treated at pH 2.0. These were the highest WCA and OCA values obtained for the LbL films prepared in this study. The aforementioned films exhibited large-scale crater-like pores along with small pores inside the craters, which indicate the importance of multi-scale hierarchical structure. However, these WCA and OCA values are lower than the values required for superhydrophobic or superoleophobic applications. For these applications, the WCA should be higher than 150° and the water rolling angle should be below 5°. However, the highest value of WCA obtained for PFTCS-coated LbL film is 9° lower, and the water droplets did not roll off the surface properly. 

**Figure 8:**
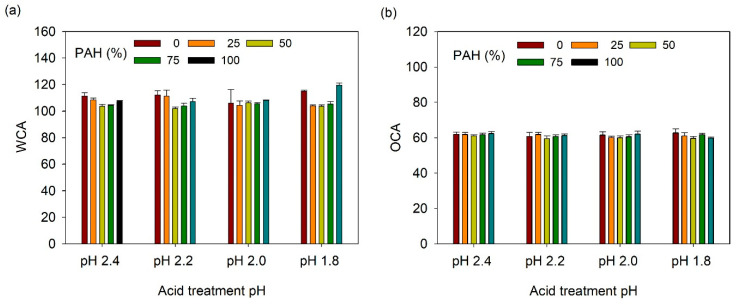
(**a**) WCA and (**b**) OCA of lubricant-infused LbL films with various PAH ratios and acid treatment pH values.

The acid-treated LbL films functionalized with TCPFS were further infused with fluorinated lubricant and their wetting properties were studied as a function of PAH ratio and acid treatment pH. First, static CA was measured (Figure 8). WCA and OCA were 105–120° and 58–62°, respectively, which are lower than those of the TCPFS coated LbL films because of the low surface energy of the fluorinated lubricant (~18 dyne/cm). However, WCA and OCA did not show a clear relationship with PAH ratio and acid treatment conditions. 

**Figure 9:**
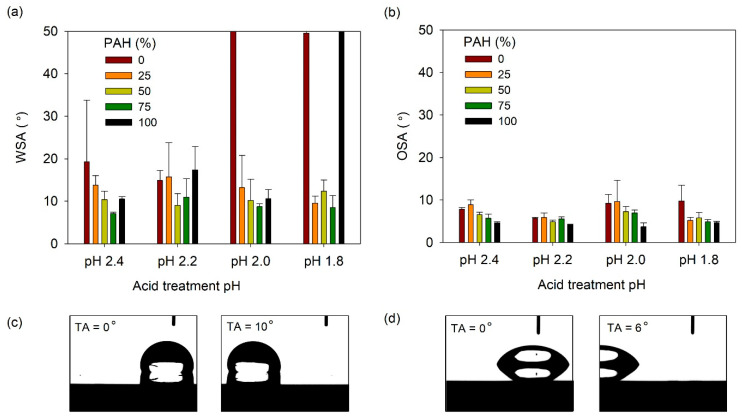
(**a**) WSA and (**b**) OSA of lubricant-infused LbL films with various PAH ratios and acid treatment pH values. A WSA of 50° denotes that the water droplet did not slide until the sliding angle increased to 50°. Optical micrographs showing the (**c**) water and (**d**) oil droplet sliding as the tilt angle (TA) increases. The lubricant-infused films of (**c**) pH 2.2, 25% PAH and (**d**) pH 2.2, PAH 50% were used.

The SA was then measured; Figure 9a shows the water slide angle (WSA) of lubricant-infused LbL films prepared at various PAH ratios and acid-treated at various pH values. Generally, the WSA was below 20°, except under the following three conditions. For the films with 0% PAH ratio treated at pH 2.0 and 1.8, the water droplet did not slide until a tilt angle of 50°. The film assembled with 100% PAH ratio, treated at pH 1.8, was not slippery. Under these conditions, the film surface did not show a clear porous structure, and the roughness was relatively low. In general, at a given pH, the films prepared with a blend of BPEI and PAH exhibited a lower WSA when compared to the films prepared with 100% BPEI or PAH. This indicates that the more complex surface morphology created from the blend of the two polymers contributes to the lower WSA of the LbL film. The lowest WA value of 7° was observed for the 75% PAH sample treated at pH 2.4. Figure 9b shows the oil slide angle (OSA) of the slippery LbL films. The OSA was less than 10° and was less affected by the acid treatment condition or the PAH ratio.

The WSA and OSA values of the lubricant-infused LbL films showed were 7–15° and 4–10°, respectively. The optimal WSA/OSA values obtained were 7.1°/5.7° and 8.6°/4.9° for films with 75% PAH at pH 2.4 and 75% PAH at pH 1.8. It is difficult to conclude which film structure exhibits the best results because of the small deviations in sliding angle among the films. However, we can conclude that films assembled with the BPEI/PAH blend exhibit sliding angles that are lower than those of films assembled with 100% PAH or 100% PAH. 

We verified the thermal stability of film wettability by measuring the CA of TCPFS-coated film after heat treatment at 125, 150, and 180 °C. WCA and OCA were stable up to 180 °C. The SA of lubricant-infused LbL films was stable up to 70 °C. The usage temperature range of the lubricant, GPL101, suggested by the manufacturer is −70 to 104 °C. Stability can be improved by using other various lubricants with higher thermal resistance.

Generally, these LbL films are regarded as amorphous glassy films. Owing to the high degree of ion-pairing, LbL films, except for hydrogen-bonded LbL films, do not exhibit T_g_ or T_m_ in differential scanning calorimetry measurements [53,54]. Further studies are needed for details.

## 4. Conclusions

This study presents various porous LbL films assembled using a BPEI/PAH blend and PAA by the acid treatment of the LbL films. Overall, the 100% BPEI film exhibited a relatively smooth surface. Below an acid treatment pH of 2.2, the films showed loss of materials, and large pores were observed beneath the skin layer above pH 2.0. Conversely, the 100% PAH film showed a microporous structure, except at pH 1.8. Microporous structures were observed at pH values of 2.4 and 2.2, and a crater-like structure with nanopores was observed at pH 2.0. At pH 1.8, the film showed a relatively smooth surface with material loss and nanopores. However, as the BPEI and PAH were blended, the films exhibited various surface textures and pore structures. Overall, the 50% PAH films showed porous structures with higher roughness when treated at pH 1.8–2.2. Particularly, the 50% PAH film treated at pH 1.8 exhibited a roughness seven times higher with a crater-like porous structure when compared to the films assembled with 100% PAH or BPEI, which exhibited a relatively smooth surface. Therefore, the different pore formation mechanisms of the BPEI/PAA film and the PAH/PAA film form various pore structures and surface morphologies when the BPEI/PAH blend is used in the LbL assembly.

The porous LbL films were coated with fluorinated silane, and the CAs were measured. At pH 2.4, the WCA increased with the increasing PAH ratio and at pH 1.8–2.0, the BPEI/PAH blend produced a synergistic effect. At pH 2.2 and 2.0, the WCA was relatively high when the PAH ratio was 50% and 100%. At pH 1.8, the WCA was the highest when the BPEI and PAH solutions were evenly blended. The dependence of the OCA on the PAH ratio and the acid treatment pH was similar to that of the WCA. The fluorinated porous LbL films were then infused with fluorinated lubricant to determine their feasibility as SLIPS. Typically, the films prepared with the BPEI/PAH blend exhibited a lower WSA than those with the pure BPEI or PAH. The films exhibited a WSA below 20°, except for three films that showed a fairly smooth surface with a porous structure. The lowest WSA value is approximately 7°. The OSA was approximately below 10° regardless of the PAH ratio and the acid treatment pH. In conclusion, acid treatment of the LbL films assembled with polyelectrolyte blends presents a novel method of obtaining various surface morphologies and pore structures, and can potentially be utilized in superwetting.

In terms of future research, the LbL films need to be further improved and analyzed for superwetting applications such as self-cleaning and anti-fouling surfaces. The TCPFS-coated LbL films need further improvement for superwetting applications, such as self-cleaning surface. Different polyelectrolyte blends and surface coating materials can be utilized to improve the superwetting performance. As for the lubricant-infused LbL films, further studies concerning their slippery performance against biological fluids or common viscous fluids such as blood or ketchup is required for specific applications such as antifouling. Moreover, the durability of slippery surfaces and their performance needs to be studied because this performance deteriorates with usage due to loss of lubricant induced by shear force.

## Data Availability

Not applicable.
